# Editorial: Women in renal pharmacology 2023

**DOI:** 10.3389/fphar.2024.1519295

**Published:** 2025-01-21

**Authors:** Nuria Lloberas, Laure Elens, Brenda de winter

**Affiliations:** ^1^ Nephrology Department - IDIBELL, Bellvitge University Hospital, Barcelona, Spain; ^2^ Louvain Centre for Toxicology and Applied Pharmacology, Institute for Experimental and Clinical Research, Université Catholique de Louvain, Brussles, Belgium; ^3^ Erasmus Medical Center, Department of Pharmacy, Rotterdam, Netherlands

**Keywords:** kidney, pharmacology, transplantation, fibrosis, immunosuppression

In the intricate dance of molecules that governs renal function and renal pharmacology, women scientists are increasingly taking the lead. These researchers, armed with knowledge and driven by passion, can enrich the field of renal pharmacology. Women are not a rare protein in the kidney, but we are underrepresented in the field, a situation that, if properly addressed, could catalyze a powerful chain reaction of innovation.

The fact that less than 30% of researchers worldwide are women highlights a persistent problem in scientific fields, including renal pharmacology. Gender biases and stereotypes continue to act as barriers, deterring girls and women from pursuing careers in STEM fields, including specialized areas such as renal pharmacology (Shen, 2013). This disparity is not merely a numbers game, but represents a significant loss of diverse perspectives, new ideas, and advances in the field of pharmacology (Nielsen et al., 2018).

However, the trend is changing. Initiatives that promote gender equality in science are gaining momentum, and organizations such as UNESCO recognize them as crucial for sustainable development. The field of renal pharmacology, with its direct impact on patient care and quality of life, can benefit greatly from greater gender diversity, essential for personalized medicine.

**FIGURE 1:**
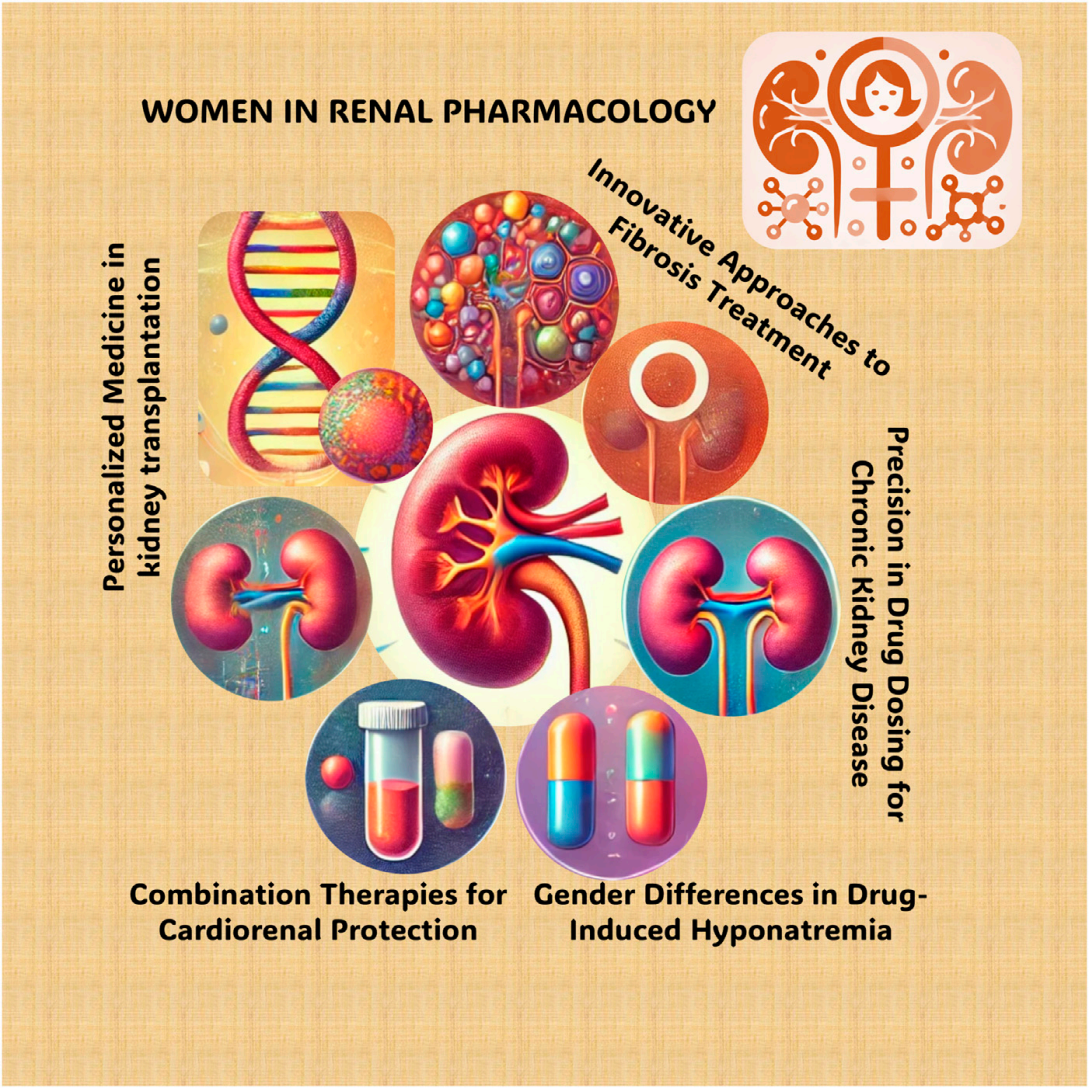
Scheme of woman in renal pharmacology fields in this editorial.

The diversity and impact of this research are exemplified by the groundbreaking studies selected for this special Research Topic (Figure 1). About Innovative Approaches to Fibrosis Treatment, a study by Saurin et al. on the macrocyclic lactone oxacyclododecindione (Oxa) demonstrates its potential as a novel therapeutic approach for fibrosis treatment. This research shows promise in reducing fibrosis progression and preventing kidney disease advancement. Concerning Precision in Drug Dosing for Chronic Kidney Disease, a study by Pluquet et al. on de-indexed estimated glomerular filtration rates (eGFRs) for oral antidiabetic drugs in chronic kidney disease patients underscores the importance of personalized dosing. This work emphasizes the need for more accurate methods to adjust drug doses, particularly in patients with extreme body mass indexes. In the field of Gender Differences in Drug-Induced Hyponatremia, critical research from Hendriksen et al., revealing that women have a higher risk of hospital admission associated with hyponatremia while using diuretics compared to men highlights the importance of sex-specific considerations in pharmacology. This study calls for further research to develop sex-specific recommendations in diuretic use. Focus on Combination Therapies for Cardiorenal Protection, the innovative work by Martos-Guillami et al., on the combined use of SGLT2 inhibitors and GLP-1 receptor agonists with RAS blockers in diabetic kidney disease models demonstrates the potential of multi-drug approaches. And finally, switching to the Personalized Immunosuppression in Kidney Transplantation, a population pharmacokinetic study on tacrolimus dosing in adult renal transplant patients’ study by Fernández-Alarcón, exemplifies the strides being made in personalized medicine. By considering factors such as CYP3A5 genotype, age, and hematocrit levels, this research provides a framework for optimizing immunosuppression regimens.

These studies collectively demonstrate the breadth and depth of women’s contributions to renal pharmacology, from basic science to clinical applications. They highlight how women-led research is driving innovation in drug development, personalized medicine, and treatment optimization across various aspects of kidney health and disease.
